# Supplementary material for: Complexome profiling of the Chlamydomonas psb28 mutant reveals TEF5 as an early PSII assembly factor
Source: Plant Cell. 2025 Mar 17;37(6):koaf055. doi: 10.1093/plcell/koaf055 (PMC12164590; doi:10.1093/plcell/koaf055)
Supplement: koaf055_Supplementary_Data [file koaf055_supplementary_data.zip › psb28_interactive.zip!/plots/intensity/qualityPlots/peptides/_Cre03.g181200.t1.2.html]

### Cre03.g181200.t1.2

Trivial name:   
SeqCoverage [median]: 3.60  
Mass: 63.81 kDa  
  
 

| Sequence | MissedCleavage | Mass [Da] | wtIntensity | muIntensity | Score |
| --- | --- | --- | --- | --- | --- |
| AGGGATAIAR | 0 | 843.46 | 505.96 | 504.14 | 144.12 |
| LVAIVANDATVK | 0 | 1212.71 | 386.78 | 515.28 | 57.60 |
|  |  |  | 892.7 | 1019.4 |  |
